# The *I2* resistance gene homologues in Solanum have complex evolutionary patterns and are targeted by miRNAs

**DOI:** 10.1186/1471-2164-15-743

**Published:** 2014-08-30

**Authors:** Chunhua Wei, Hanhui Kuang, Feng Li, Jiongjiong Chen

**Affiliations:** Key Laboratory of Horticulture Biology, Ministry of Education, and Department of Vegetable Crops, College of Horticulture and Forestry Sciences, Huazhong Agricultural University, Wuhan, 430070 People’s Republic of China

**Keywords:** *I2* locus, *Ty-2*, Solanaceae, Evolution, Sequence exchange, miRNA

## Abstract

**Background:**

Several resistance traits, including the *I2* resistance against tomato fusarium wilt, were mapped to the long arm of chromosome 11 of Solanum. However, the structure and evolution of this locus remain poorly understood.

**Results:**

Comparative analysis showed that the structure and evolutionary patterns of the *I2* locus vary considerably between potato and tomato. The *I2* homologues from different Solanaceae species usually do not have orthologous relationship, due to duplication, deletion and frequent sequence exchanges. At least 154 sequence exchanges were detected among 76 tomato *I2* homologues, but sequence exchanges between *I2* homologues in potato is less frequent. Previous study showed that *I2* homologues in potato were targeted by miR482. However, our data showed that *I2* homologues in tomato were targeted by miR6024 rather than miR482. Furthermore, miR6024 triggers phasiRNAs from *I2* homologues in tomato. Sequence analysis showed that miR6024 was originated after the divergence of Solanaceae. We hypothesized that miR6024 and miR482 might have facilitated the expansion of the *I2* family in Solanaceae species, since they can minimize their potential toxic effects by down-regulating their expression.

**Conclusions:**

The *I2* locus represents a most divergent resistance gene cluster in Solanum. Its high divergence was partly due to frequent sequence exchanges between homologues. We propose that the successful expansion of *I2* homologues in Solanum was at least partially attributed to miRNA mediated regulation.

**Electronic supplementary material:**

The online version of this article (doi:10.1186/1471-2164-15-743) contains supplementary material, which is available to authorized users.

## Background

Most of the disease resistance genes cloned from plant species encode nucleotide-binding site (NBS) and leucine-rich repeat (LRR) domains. The NBS-LRR encoding genes are often called disease resistance genes (or *R*-genes), since their main functions are protecting plants from pathogens with only a few exceptions, such as CHS3, a TIR-NBS-LRR-LIM encoding gene, playing role in cold stress and ADR1, a CC-NB-LRR encoding gene, involved in drought tolerance [1–4]. The *R*-genes belong to a large gene family, with dozens or hundreds of copies in a genome [5–7]. The R proteins are composed of a variable N terminus, a conserved central NBS domain and a C-terminus with various number of short LRR motifs [8]. The N terminus usually has a Toll/interleukin-1 receptor (TIR) motif or a coiled-coil (CC) motif [9, 10]. The NBS domains can bind and hydrolyze ATP or GTP, which are involved in signaling leading to pathogen resistance [11]. The LRR domains are most likely to be involved in recognition specificity [12, 13].

*R*-genes tend to be clustered in plant genomes [6, 9]. Many *R*-gene sub-families were shown to have been under diversifying selection, particularly at the hyper-variable solvent-exposed residues in the LRR region [14, 15]. Consequently, *R*-genes represent the most divergent gene families in plant genomes [16]. Besides diversifying selection, frequent sequence exchanges among homologues have also contributed to the high divergence of *R*-gene families in plants [17, 18]. However, *R*-genes vary dramatically in frequency of sequence exchanges. Some *R*-genes, termed Type I *R*-genes, had frequent sequence exchanges with homologues, resulting in extensive chimeric structures [15, 18]. Frequent sequence exchanges may completely abolished allelic relationship of Type I *R*-genes from different genotypes. Different genotypes of the same species may harbor a various set of chimeric *R*-genes (Type I), leading to enormous distinct *R*-gene sequences in a population [19]. In contrast to the Type I *R*-genes, some *R*-genes (termed Type II) do not recombine with paralogues and are highly conserved in different genotypes of a species or closely related species. However, some Type II *R*-genes were found to be frequently lost in some genotypes, exhibiting presence/absence polymorphism [6, 20]. The mechanism underlying the differentiation of above two distinct evolutionary patterns for *R*-genes remains unknown.

The resistance gene *I2* in tomato (*Solanum lycopersicum*) encodes resistance against race 2 of the fusarium wilt pathogen *Fusarium oxysporum* f sp *lycopersici* [21]. The *I2* gene is a member of a gene cluster located on chromosome 11 of tomato. The I2 protein is a typical CC-type R protein. A resistance trait against tomato yellow leaf curve virus (TYLCY) was also mapped in the vicinity of the *I2* locus though it remains unclear whether the trait is encoded by a *I2* homologue [22, 23]. *I2* homologues are found in the syntenic region of potato (*S. tuberosum*); two resistance genes (*R3a* and *R3b*) against potato late blight have been cloned from this region of potato [24, 25]; and at least nine additional *R*-genes against potato late blight were mapped to this locus, which harbors dozens of *I2* homologues [24, 26, 27]. The *R3a* and *R3b* from potato exhibit 88% and 80% nucleotide identity with the *I2* gene from tomato,respectively. *I2* homologues were also found at the corresponding region in pepper (*Capsicum annuum*), and this locus may represent the most important *R*-gene locus in Solanaceae [28]. Previous studies of the *I2* locus mainly focused on the cloning of functional *R*-genes, but the structure, evolution and gene content of the entire cluster remain unclear.

*R*-genes are believed to be physiologically toxic to plant cells and their expression is usually kept at low level when no pathogens exist [29, 30]. Some *R*-genes are up-regulated when challenged by pathogens [31]. One mechanism to keep *R*-gene at low level in absence of pathogen is through miRNA mediated gene silencing [32, 33]. Some *R*-genes could be cleaved by 22-nt miRNAs and successfully produce secondary small interfering RNAs (tasiRNAs) in a phased fashion (phasiRNA). The resistance gene *R3a* from potato was shown to be cleaved by 22-nt miR482 family and produce phasiRNA [33]. Whether the *I2* family from tomato is regulated by miR482 or other miRNAs remains unknown.

In this study, we analyzed the structure and evolution of the *I2* locus in tomato, and compared it with that in potato. First, *I2* homologues were cloned from several genotypes of cultivated and wild tomato, including genotypes with resistance against TYLCV. Their evolution was investigated in detail through sequence analysis of *I2* homologues from multiple genotypes of tomato, potato, pepper and tobacco. The miRNAs targeting *I2* homologues in potato and tomato were investigated and compared. The role of miRNAs on *R*-gene expansion was discussed. The comprehensive study of the structure of the *I2* locus may facilitate the cloning and the use of *R*-genes located in this region, and the evolutionary study may shed light on the mechanism of *R*-gene expansion in a genome.

## Methods

### Tomato materials used in this study

Since resistance gene *Ty-2* against TYLCV was genetically mapped to the *I2* locus, a commercial tomato hybrid cultivar Hongxiaoli with *Ty-2* was included in this study. The hybrid was selfed and a F2 population with 736 individuals was generated. A homozygote *Ty-2/Ty-2* (flanking markers are homozygous) was obtained from the F2 population to represent the *Ty-2* (T) haplotype. Similarly, a susceptible homozygote of *ty-2* was identified to represent the susceptible ty-2 (t) haplotype. The F2 population was used to map *I2* homologues from the T and t haplotypes. The T haplotype was introgressed into cultivated tomato from *S. habrochaites* [23, 34]. Four additional *S. habrochaites* accessions, LA1777, LA1740, LA2158 and LA2860, were also included in this study for *I2* homologue analysis.

### Inoculation of TYLCV and phenotyping

To phenotype above tomato genotypes on reaction to TYLCV, seedlings of 4–6 leaf stages were inoculated with a TYLCV agrobacterium infectious clone [35]. Inoculated seedlings were kept in a growth chamber under 16 h light/8 h dark cycle for a month before investigating symptom of TYLCV (curve leaf).

### PCR amplification, cloning and sequencing of *I2*homologues

The tomato genomic DNA were extracted from mature leaves of each genotypes using CTAB method, and genomic DNA were used as template to PCR amplify *I2* homologues [36]. Nearly full-length (~3.5 kb) fragments of *I2* homologues were amplified from aforementioned genotypes of tomato using a pair of degenerated primers (Table 1), which were located at +165 and +3,685 of the resistant gene *I2*, respectively. PCR amplification was in a 25 μl reaction with 1 unit Fast Pfu Taq (TransGen, Beijing, China), treated for 5 mins at 95°C, followed by 32 cycles at 95°C for 30 s, 52°C for 30 s, and a final extension at 72°C for 1.5 min. PCR products were gel purified using Gel Purification Kit (Generay, Shanghai, China) and ligated into vector pZERO5 using TA cloning kit (TransGen, Beijing, China). Individual colonies were sequenced until no new genes were obtained in the last ten colones. If sequences from different clones have higher than 99.7% nucleotide identity, they were considered to be derived from the same gene. The *I2* homologues amplified from an accession were named as accession name followed by “*I2*” then by a number, such as *LA1777-I2-1. I2* homologues from the T and t haplotypes were named as *T-I2-* and *t-I2-* followed by a number, respectively.

**Table 1:** **Primer sequences used for this study**

Marker	Sequences 5′to 3′	Note
M-73000	F:ATTCCCACCCTTGATGATGT	Screening recombinant individuals
	R:TTCTTTAGCCAACTCCTTGC	Restriction enzyme: Taq I
M-137	F:TAGCTTGGGATCGACATCTT	Screening recombinant individuals
	R:CTCGTTCTCGCATTCATTTA	Restriction enzyme: Mbo I
I2	F:TGTGCTAAGTGAYGCASAGA	Amplification of *I2* homologs
	R:TAGAGAGGGRAGRGCA	
T-I2-1	F:GCTTGAATTTAGAGATATGCG	Mapping *I2* homologs
	R:GCTTAGGGCAATAGAAGATAGT	
T-I2-5	F:CCCTTCTACAAATTGGAGCACA	Mapping *I2* homologs
	R:GGTAGTTTGCAGTATGCTAACA	
T-I2-8	F:ACCACTGACTGTTACTTTCTAT R:ATTAGGGCAATGGTAGATCAC	Mapping *I2* homologs
T-I2-2	F:TTCTCCTGTCATCTTGTTGTTC	Mapping *I2* homologs
	R:CCAAGTAACGTTCTGGCAATTT	
T-I2-3	F:GGCTTAATGGGCTTCGAGTC R:GTTCCACAAGTGACGGTAT	Mapping *I2* homologs
T-I2-6	F:AGATTGAAAGCATTAAATATCA	Mapping *I2* homologs
	R:ATTGGATACCTCAAGTCTTGT	
T-I2-7	F:GTGGCTTAATGAGCTTCGAGA	Mapping *I2* homologs
	R:TCACATTCTACCACTTTCAAG	
T-I2-9	F:CCAATCCAGTTCACTCTTTCA	Mapping *I2* homologs
	R:ACCAGTTTCTTGCAATCATA	
T-I2-10	F:GTCCCAAATCCTTCAGAG	Mapping *I2* homologs
	R:CAAAGATTTTCAGGCAACTTT	
Race-P1	R: GGAAGATCATTGTAGCTCAACATYA	Identification of miRNAs’ cleavage sites
Race-P2	R: CGTGTMGTCACAATGATCTTACTTCC	Identification of miRNAs’ cleavage sites
Race-P3	R: TTTCCTTCAATTTGACTTGWAGC	Identification of miRNAs’ cleavage sites

The usage of primers is listed in ‘Note’ column. Restriction enzyme indicates the marker is a CAPS marker.

### Sequence analysis

Four *I2* homologues [*I2* [GenBank: AF118127], *I2C-1* [AF004878], *I2C-2* [AF004879], *I2C-5* [AF408704]], originated from wild tomato species *S. pimpinellifilium* were downloaded from GenBank [21, 37, 38]. Two partial genes *I2C-3* and *I2C-4* were excluded from this study. The sequences of *R3a* [AY849382], *R3b* [JF900492.1] and sixteen *I2* homologues [AY849383-AY849385, EF638450-EF638453, EF638455, EF638456, EF638458, EF638460-EF638465] from potato genotype SH83-92-488 were retrieved from GenBank and named as *SH-I2-* followed by a number [24, 25]. The *I2* homologue [HQ731036] from *S. bulbocastanum* was named as *SB-I2-1*, and the one [HQ731037] from *S. stoloniferum* as *SS-I2-1*.

Using BLASTN method, *I2* homologues (>2,500 bp) were retrieved from the sequenced tomato genotype Heinz 1706 [39] and the sequenced potato genotype DM [40]. They were named as *HZ-I2-* and *DM-I2-* followed by a number, respectively. Sequences were aligned using program Muscle [41] and manually edited in GeneDoc (http://www.nrbsc.org/gfx/genedoc/). Neighbor-joining (NJ) trees (Kimura two-parameter substitution model) with bootstrap values (1,000 replications) were constructed using Mega 5.0 [42]. Gene conversions among homologues were detected using Geneconv [43] with the default settings and confirmed visually. Dot plot analysis between two sequences was performed using program DOTTER [44].

### Mapping of the *I2*homologues in the T and t haplotypes

A F2 population with 736 individuals was used to map the *I2* homologues from the T and t haplotypes. First, primers specific to each *I2* homologue were designed (Table 1). A primer pair was considered as specific only when the target gene but none of the other genes in the T and t haplotypes was amplified by this primer combination. The gene-specific primers were used as markers to screen the F2 population and their linkage with CAPS markers M-73000 and M-137 at the *I2* locus were analyzed.

### Identification of miRNAs targeting *I2*homologues

To detect miRNAs that potentially target *I2* homologues, sequences of all *I2* homologues were first used as query to screen for matching small RNAs (sRNA) from database SoMART (http://somart.ist.berkeley.edu/) [45]. Identified sRNAs with more than 100 reads in the database were further confirmed in the psRNAtarget web server (http://plantgrn.noble.org/psRNATarget) using following parameters: maximum expectation = 5.0, and target accessibility - allowed maximum energy to unpair the target site (UPE) = 50. All confirmed matching sRNAs were mapped to tomato genome using bowtie [46]. Then, approximately 800 bp flanking sequences of all confirmed matching sRNAs were extracted, and their fold-back structures were predicted using the RNAfold program with default settings [46, 47]. A folding structure was assumed if there was a central loop and a stem (matching region) of at least 18 bp when folding energy no greater than 18 kcal/mol was used. Then, the fold-back structures were evaluated using MirCheck program with strict parameters: ≤4 mismatches, ≤2 bulged or asymmetrically unpaired nucleotides and ≤2 continuous mismatches in the seed regions to meet the accepted criteria for miRNA annotation [48, 49].

The genomes of *S. lycopersicum* (ITAG version 2.3), *S. tuberosum* (version 3_2.1.11), *Nicotiana benthemiana* (version 0.4.4), *N. sylvestris* (GenBank Assembly ID: GCA_000393655.1), *N. tomentosiformis* (GenBank Assembly ID: GCA_000390325.1), *Capsicum annuum* (version 2.0), *Mimulus guttatus* (version 2.0), *Vitis vinifera* (Phytozome v9.0: Vvinifera_145), *Carica papaya* (Phytozome v9.0: Cpapaya_113) and *Arabidopsis lyrata* (GEO accession: GSE45676) were chosen to analyze if they contain a certain miRNA. First, a mature miRNA sequence was downloaded from miRBase (release 20), then mapped into these genome sequences using bowtie. If a genome sequence had no more than 4 mismatches with a miRNA sequence, its 800 bp flanking sequences were further investigated for stem-loop structure using program RNAfold and evaluated using program MirCheck as described above.

Three miR6024 hairpin sequences (tomato, potato and tobacco) were downloaded from miRbase (release 20). They were used as query sequence in BLASTN search of ten plant genomes, and significant hits (E value cutoff of 1e^−10^) were retrieved for further analysis.

### Mapping of mRNA cleavage sites in *I2*homologues

Total RNAs were isolated from leaves of tomato Hongxiaoli plantlets using RNAiso Plus (Takara, Dalian, China) and was purified with RNA cleanup Kit (Kangwei, China). To map the miRNA cleavage site in *I2* homologues, modified 5′- RACE was performed using GeneRacer Kit (Invirogen, Carlsbad, CA) as described previously [50]. First, approximately 5 μg total RNA was ligated with the GeneRacer RNA Oligo adapter (250 ng). Then, GeneRacer Oligo dT primer or conserved *I2* primer was used to synthesize the first strand cDNA [51]. A degenerate primer (Race-P1) was designed in regions conserved in most tomato *I2* homologues to pair with GeneRacer™ 5′ primer to amplify PCR products using the synthesized cDNA as template. Then, GeneRacer™ 5′ nest primer and gene nested primers, Race-P2 and Race-P3, were used for the first round nested PCR and second round nested PCR, respectively (Table 1). The PCR products were gel purified using Gel Purification Kit (Generay, Shanghai, China) and ligated into vector pEASY-T1 using TA cloning kit (TransGen, Beijing, China). Positive clones were sent for sequencing.

Cleavage sites of tomato *I2* homologues were also investigated using a degradome database using online program SoMART [45]. First, a randomly chosen tomato *I2* homologue was input into programs Silcer Detector and dRNA mapper. The output of these two programs was then analyzed by a third program SMART COMPAR.

## Results

### The *I2*locus spans more than 5 Mb on chromosome 11 of tomato and potato

**Figure 1:**
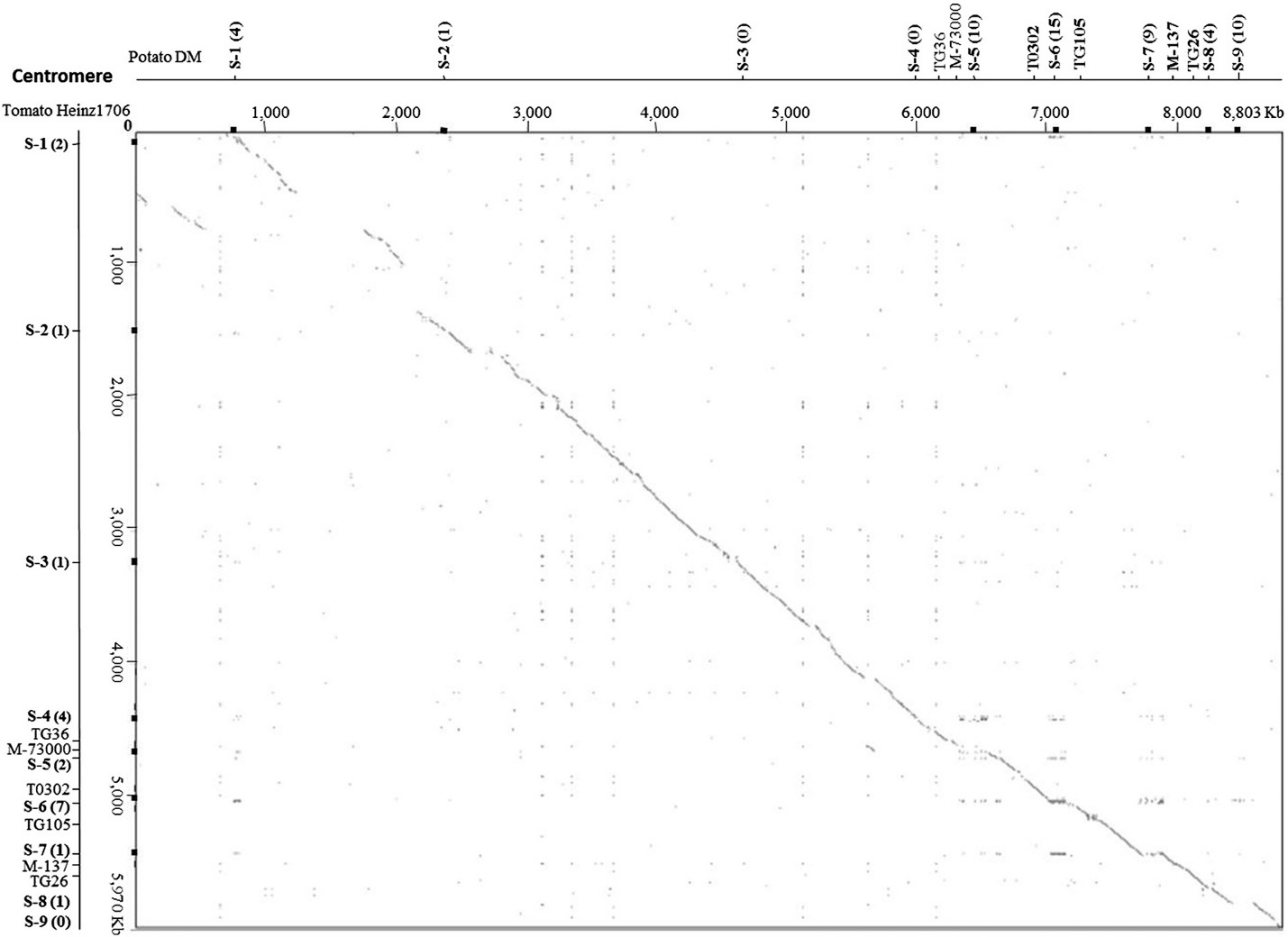
**Dot plot analysis of the** ***I2*** **locus between tomato and potato.** The vertical line represents the *I2* locus in tomato cultivar Heinz 1706, and the horizontal line represents the *I2* locus in potato cultivar DM. The positions for nine sub-loci (S-1 to S-9) and important markers are shown in both the horizontal and vertical lines. The numbers in brackets after the sub-locus name are the number of *I2* homologues at corresponding positions. No large duplications were found, and duplications were mainly limited to the *I2* sequences but not their flanking regions.

Using BLASTN method, 36 *I2* homologues were discovered in the sequenced genome of tomato cultivar Heinz 1706. Nineteen of them are located at the *I2* locus, in a 5.4 Mb region on chromosome 11. Of the remaining 17 homologues, five are from other chromosomes and 12 are on chromosome 11 but away from the *I2* locus, and they were excluded from further analysis (Additional file 1: Table S1). The positions of *I2* homologues and important genetic markers around the *I2* locus of tomato Heinz 1706 are shown in Figure 1.

The sequenced genome of potato DM has 71 *I2* homologues. Of them, 53 are located in the syntenic region of the *I2* locus of tomato, spanning a region of at least 7.4 Mb (Additional file 1: Table S1). Fifteen *I2* homologues are not on chromosome 11, and three *I2* homologues are on chromosome 11 but far away from the syntenic region of the *I2* locus. Therefore, the *I2* locus was most likely expanded before the speciation of Solanum and may have also experienced amplifications after speciation.

### Comparative analysis of the *I2*/*R3*locus in tomato and potato

To investigate structure variation between tomato and potato, the *I2* locus of tomato Heinz 1706 was compared with that of the potato genotype DM. Good co-linearity was found between tomato and potato, except in the centromeric part of the locus. However, the copy number of *I2* homologues as well as their positions varies considerably between the two genomes (Figure 1).

Based on the distribution patterns of *I2* homologues, this locus can be further divided into 9 sub-loci (sub-clusters). Dot plot analysis indicated that the duplications resulting in the 9 sub-loci were limited to the *I2* sequences but not their flanking regions, since sequences flanking these *I2* homologues are unrelated (Additional file 2: Figures S1 and S2). *I2* homologues are presented at six sub-loci of both tomato and potato genomes, though their copy number may vary dramatically (Figure 1). At the other three sub-loci (sub-loci 3, 4 and 9), *I2* homologues are present in one but absent in the other genome, showing presence/absence divergence between the two species.

**Figure 2:**
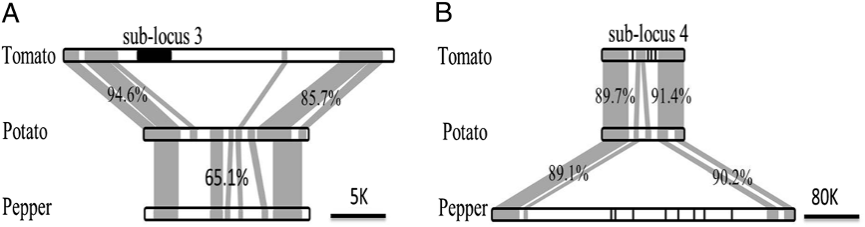
**Duplications and deletions of** ***I2*** **homologues.** Shaded region represents homologous sequences between two species, and the percentage refers to nucleotide identity between the two sequences. **A.** *I2* homologue (black box) is present in tomato but absent in potato and pepper, suggesting that the *I2* homologue at sub-locus 3 in tomato was gained after speciation. **B.** *I2* homologues (black vertical lines) are present in tomato and pepper but absent in potato, suggesting that the *I2* homologue at sub-locus 4 in potato were lost after speciation.

To investigate the genetic mechanism for such presence/absence divergence at the three sub-loci, their sequences were used to search the pepper genome. Sequences from the syntenic regions of sub-loci 3 and 4 but not 9 were found in pepper genome. Like in potato, the sub-locus 3 in pepper does not have any *I2* homologues (Figure 2A), suggesting that the *I2* homologue at sub-locus 3 of tomato was most likely gained after speciation. On the other hand, potato might have lost *I2* homologues at sub-locus 4 after speciation since pepper has *I2* homologues at this sub-locus (Figure 2B).

To study the evolution of *I2* homologues from different sub-loci, a distance tree was constructed using approximately 2.5 kb sequences of *I2* homologues (their 3′ parts were excluded due to various duplications) from tomato (16), potato (39), pepper (9) and *N. sylvestris* (18) and *N. tomentosiformis* (11) (Additional file 2: Figure S3). Homologues *HZ-I2-5* and *DM-I2-22*, which contain many unknown nucleotides, were excluded from further analysis. Based on the tree, the *I2* homologues from Solanaceae can be classified into four major groups. One group contains *I2* homologues from sub-loci 4 and 5 of pepper; one group contains all *I2* homologues from sub-loci 1–7 of tomato and potato; and the other two groups contain *I2* homologues from sub-loci 8 and 9 of potato and pepper as well as all *I2* homologues from Nicotiana. The phylogenetic tree suggests that all *I2* homologues from Nicotiana might be derived from sub-loci 8 and 9 in the common ancestor between Solanum and Nicotiana. The *I2* homologues from sub-loci 1 and 2 of tomato are highly similar to those of potato, respectively. However, homologues in other sub-loci showed no obvious orthologous relationship between tomato and potato. The lack of orthologous relationship between *I2* homologues in tomato and potato suggest that sequence exchanges may have occurred between homologues from different sub-loci after speciation of Solanum.

### Sequencing *I2*homologues from different genotypes

To better understand the diversity and evolution of the *I2* locus, *I2* homologues were cloned from six tomato genotypes, including the T and t haplotypes (see MM section), and four wild genotypes of *S. habrochaites* (LA1777, LA1740, LA2158 and LA2860). Inoculation with the infectious TYLCV clone showed that LA1777 is resistant to TYLCV, consistent with previous conclusion [52], while the other three wild genotypes are susceptible. To clone the *I2* homologues from these genotypes, a pair of degenerated primers (I2-F/R) was designed from the conserved regions of *I2* homologues (Table 1). Their PCR products were cloned and individual colonies were sequenced. A total of 75, 83, 48, 55, 70 and 70 colonies were sequenced, resulting in 8, 11, 13, 6, 13 and 11 distinct sequences from the T and t haplotypes, *S. habrochaites* accessions LA1777, LA1740, LA2158 and LA2860, respectively.

### Mapping of the *I2*homologues in the T and t haplotypes

**Figure 3:**
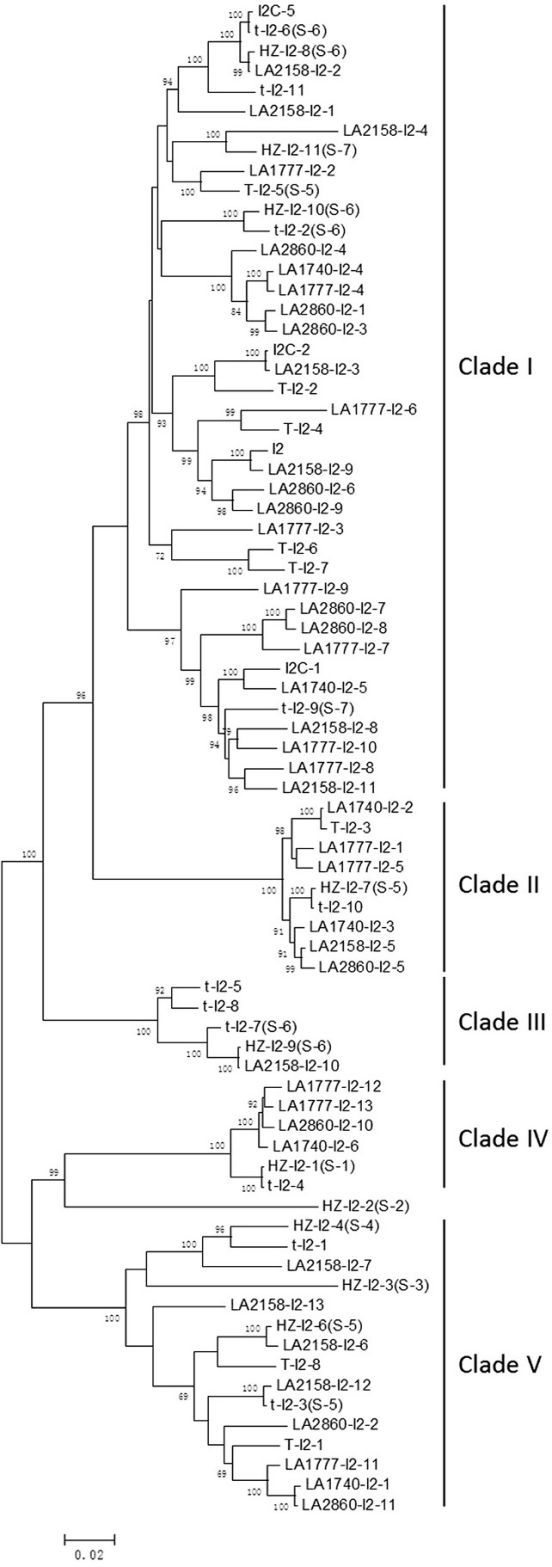
**Distance tree of** ***I2*** **homologues from different genotypes of tomato.** The 76 *I2* homologues from tomato form five clades, supported by high bootstrap numbers. Members in clades I, III and V are evenly related homologues, and are extensive chimeras. Members within clades II and IV are highly conserved homologues from different genotypes. The first part of the gene name represents accession/cultivar name, and the numbers in bracket (if any) show gene’s position (sub-locus). Genes from the same genotype spread in the tree. Numbers on nodes are bootstrap values, and values <65 are not shown.

The 8 and 11 *I2* homologues obtained from the T and t haplotypes were genetically mapped using a F2 segregating population (736 individuals) derived from the hybrid cultivar Hongxiaoli. First, markers M-73000 and M-137, which flank the *I2* locus, were used to screen for recombinants. A total of 44 recombinants were obtained from the 736 F2 individuals. Specific primers were successfully designed for six of the 19 *I2* homologues from the T and t haplotypes, and all of them were fine mapped to sub-loci 5–7 of the *I2* locus (Figure 3).

### Different evolutionary patterns for *I2*homologues

The 62 nearly full-length *I2* homologues obtained above were combined with the 10 *I2* homologues (>2.5 Kb) from Heinz 1706 and four homologues from wild species *S. pimpinellifilium* for further analysis [Genbank: KJ652840 – KJ652901] (Additional file 1: Table S1). A distance tree of the 76 *I2* homologues showed five distinguishable clades (Figure 3). Members from clade II and IV are highly conserved, with all pair-wise nucleotide identities >96.9%. Each genotype/haplotype usually has one representative in clades II and IV, and members within each clade are most likely alleles/orthologues. The exceptions are two *I2* homologues from LA1777 in Clade IV, and two *I2* homologues from LA1777 and LA1740 in clade II, respectively. The presence of two highly similar sequences is most likely due to the heterozygosity of the *I2* locus in these genotypes, which are self-incompatible. Only a few sequence exchanges were detected in clades II and IV, and mainly occurred between alleles (Additional file 1: Table S2). Therefore, clades II and IV have evolved independently from other homologues at the *I2* locus, with an evolutionary pattern similar to that of Type II *R*-genes [15].

In contrast, the other three clades in Figure 3 usually have multiple copies from one genotype and the average pair-wise nucleotide identity within a clade varies from 91.3% to 96.6%. Homologues within a clade are often equally related with each other and do not show obvious allelic/orthologous relationship. High sequence diversity and lack of allelic/orthologous relationship might have been attributed to frequent sequence exchanges among homologues [15]. To test above hypothesis, sequence exchanges were analyzed for all *I2* homologues in tomato using software Geneconv, and a total of 154 sequence exchanges were detected. The length of the sequence exchange tracts varied from 77 to 3,022 bp, with an average of 533 bp. Of them, 143 sequence exchanges occurred among members within a clade (98 within clade I, 7 within clade II, 8 within clade III, 1 within clade IV and 29 within clade V) (Additional file 1: Table S2). Clade I has homologues from three sub-loci (5, 6 and 7), and sequence exchanges happened between homologues from different loci. The frequent sequence exchanges and extensive chimeric structures suggest that the *I2* homologues in clades I and V have an evolutionary pattern of Type I *R*-genes [15].

### Sequence exchanges occasionally occurred between Type I and Type II *I2*homologues or between different lineages of Type I genes

Eleven sequence exchanges were detected between genes from different clades. A sequence exchange of 162 bp was detected between *LA1740-I2-5* from clade I and three members (*LA1740-I2-3, LA2158-I2-5, LA2860-I2-5*) from clade II (Additional file 1: Table S2 and Additional file 2: Figure S4). The three members from clade II have identical sequences in the exchange tract. It is unlikely that the three genes had independent sequence exchanges in the same region. It is most likely that a 162 bp sequence of *LA1740-I2-3* converted gene *LA1740-I2-5*, since these two genes are from the same genotype (LA1740). Similarly, gene *LA1777-I2-1* from clade II converted gene *t-I2-2* from clade I. Sequence exchanges might also occur between different lineages of Type I genes, such as between genes *HZ-I2-10* from clade I and *t-I2-5* from clade III. Interestingly, the directions of above sequence exchanges were unilaterally from Type II genes to Type I genes or between different lineages of Type I genes, but never from Type I genes to Type II genes.

### Rare sequence exchanges among *I2*homologues in potato

**Figure 4:**
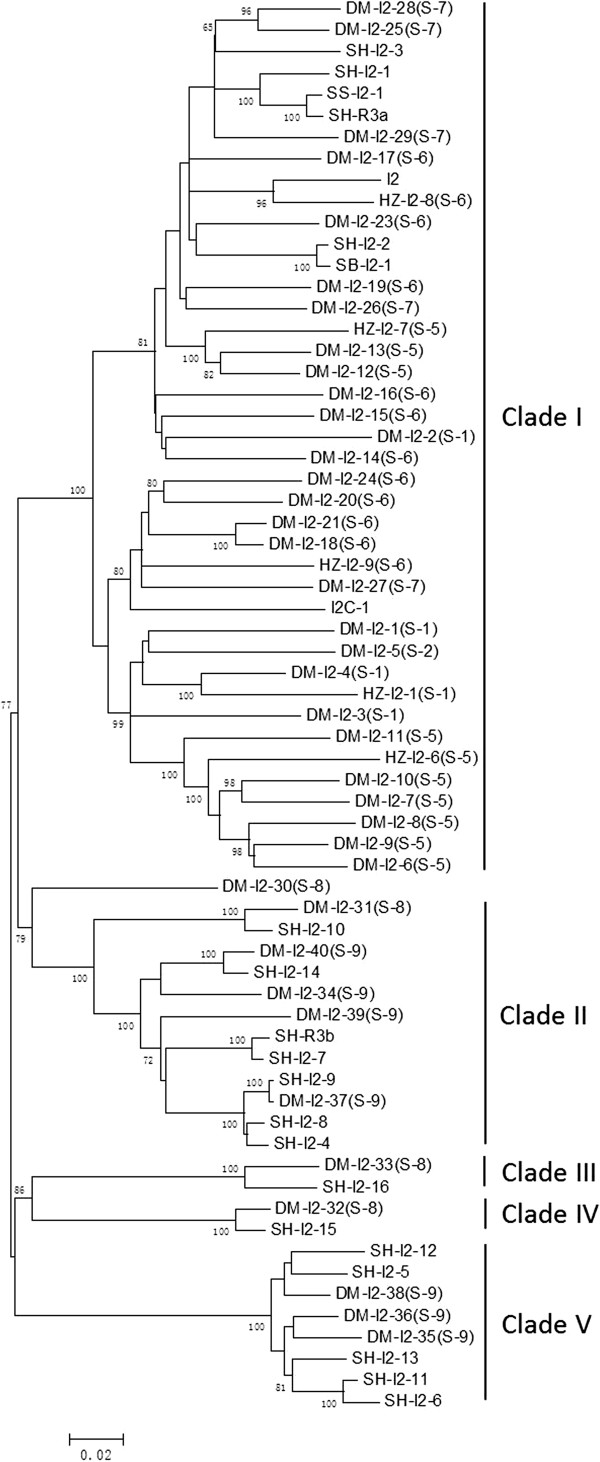
**Distance tree of** ***I2*** **homologues from potato.** Seven representative *I2* homologues from tomato are included, which are all grouped into clade I. Genes with name “*DM-I2-*” are from potato cultivar DM; genes with name “*SH-I2-*” are from *S. tuberosum*; genes with name “*HZ-I2-*” are from tomato Heinz 1706. Numbers on nodes are bootstrap values, and values <65 are not shown.

Similar analyses (including phylogenetic analysis and sequence exchange) were applied to *I2* homologues in potato. A total of 63 *I2* homologues (>2.5 Kb, and one gene with many unknown nucleotides excluded) were obtained from Genbank, mainly from the sequenced genome of DM and the *R3a* haplotype of the diploid *S. tuberosum* (Additional file 1: Table S1). Three of them are not from the *I2* locus and one has too many missing data, and they were excluded from further sequence analysis. A distance tree was constructed for the remaining 59 potato *I2* homologues, and seven tomato homologues including *I2, I2C-1* and one from each clade in Figure 3 were included for comparison. Consistent with their locations shown in Figure 1, all *I2* homologues from tomato are grouped together with homologues from sub-loci 1–7 of potato (clade I in Figure 4). The homologues from clade I in Figure 3 are quite divergent (mostly < 90% nucleotide identity, with an average of 87.5%) but evenly related. Seven sequence exchanges were detected among the 34 *I2* homologues from potato in clade I (Additional file 1: Table S2). Such pattern is in striking contrast to that for the *I2* homologues from tomato, in which *I2* homologues were differentiated into several groups, including both Type I and Type II genes (see above). No obvious differentiation (i.e. no well supported clades in Figure 4) among potato *I2* homologues and occasional sequence exchanges suggest that the *I2* homologues in sub-loci 1–7 in potato represent an ancient lineage of Type I genes that were originated at least 7 MYA before the divergence of tomato and potato [53, 54].

The homologues from sub-loci 8 and 9 of potato form four clades (II, III, IV and V) in Figure 4. Ten and two sequence exchanges were detected among members in clade II and clade V, respectively. Members within these two clades exhibited 91.8% and 95.3% average nucleotide identity, respectively. Sequence exchanges among members of each clade and high diversity of *I2* homologues suggest that clades II and V represent two distinct lineages of Type I *I2* homologues [15]. The evolutionary pattern of clade III and IV remains unclear because only two sequences were obtained from this study.

### Identification of miRNAs for the *I2*homologues

**Figure 5:**
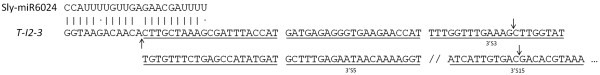
**Cleavage sites of miR6024 and two tasiRNAs in gene** ***T-I2-3*** **.** The 22 nt miR6024 has three mismatches with gene *T-I2-3*. The blocks of 21 nt (marked by underlines) show the tasiRNAs triggered by miR6024, and the 6^th^-14^th^ tasiRNAs were omitted as indicated by “//”. The arrow in *T-I2-3* matching the miR6024 sequence shows the cleavage site of miR6024, evidenced by RACE-PCR and degradome analysis. The arrows in the third (3′S3) and 15^th^ (3′S15) tasiRNAs are most likely the cleavage sites of tasiRNAs*.*

The resistance gene *R3a* from potato was shown to be targeted by members of 22-nt miR482 family [33]. Computational analysis showed that *I2* homologues from tomato may also be targeted by miR482 family, which has about 380 reads per million from three tissues (leaf, flower and fruit) of tomato (miRbase release 20). To investigate if *I2* homologues from tomato are targeted by other miRNAs, the tomato sRNA libraries (http://somart.ist.berkeley.edu) were first searched using 76 *I2* homologues. Consequently, a total of 1,439 distinct sRNAs, including the 22-nt sly-miR6024, were found in the sRNA database when five mismatches were allowed. Sly-miR6024 was previously shown to regulate the expression of *R*-gene *Tm-2* in tomato [33]. To investigate if sly-miR6024 also target the *I2* homologues, modified RNA ligase-mediated 5′-RACE was performed [50, 51]. Sequencing the PCR products showed that the mRNAs of at least one *I2* homologue from cultivar Hongxiaoli were cleaved at the predicted targeting site of miR6024 (Figure 5 and Additional file 2: Figure S5). However, no cleavage product was detected at the predicted target site of miR482. Therefore, the *I2* homologues from tomato are targeted by miR6024 and may not be targeted by miR482. The cleavage of miR6024 on *I2* homologues was also confirmed in a degradome database of tomato. Fourteen partial *I2* cDNAs were identified in the database. One of partial ones starts from the 485^th^ nucleotide of gene *T-I2-3*, confirming the cleavage function of miR6024 (Additional file 1: Figure S6A).

The targeting site of sly-miR6024 encodes the 206^th^ – 213^th^ amino acids in the I2 protein, partially overlapping with the conserved P loop. This sequence is highly conserved in all *I2* homologues in tomato and potato. We hypothesize that miR6024 regulate expression of most, if not all *I2* homologues in tomato, which may facilitate its expansion in a genome.

### MiR6024 triggers 21-nt phased siRNA from *I2*homologues

It was shown that 22-nt miRNAs often trigger the biogenesis of secondary phased siRNA [55, 56]. To investigate if miR6024 can trigger phasiRNA, small RNAs from tomato were analyzed using program SoMART [45]. A total of 847 sRNAs were successfully mapped to a representative *I2* homologue (*T-I2-3*). They were predominantly 21-nt in length and with 5′ U residue, consistent with the features of tasiRNA [33, 57]. Most of them were mapped to the downstream of the cleavage site of miR6042 with a phased pattern (Additional file 2: Figure S6B). The structure of these sRNAs and their phasing with the miR6024 cleavage site indicated that they were tasiRNAs triggered by the 22-nt miRNA6024.

RACE-PCR and degradome database were used to investigate the potential regulating effects of tasiRNAs. Three potential cleavage sites of phasiRNA were identified. A degraded mRNA of gene *T-I2-6* starting at 579^th^ nucleotide and a degraded mRNA of gene *T-I2-3* starting at 540^th^ nucleotide, were obtained using RACE-PCR strategy (Additional file 2: Figure S6C). These two degraded mRNA were most likely the cleavage products of tasiRNAs (3′S5 and 3′S3, respectively) since their cleavage points are located in the middle of a tasiRNA triggered by the miRNA6024 (Figure 5). In addition, a degradome RNA from tomato degradome database was most likely generated by phasiRNA 3′S15 (Figure 5 and Additional file 2: Figure S6D).

### MiR6024 was originated after the divergence of Solanaceae

**Figure 6:**
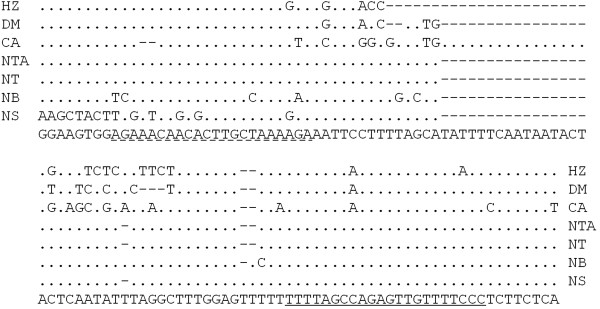
**Alignment of the miR6024 genes in Solanaceae.** The bottom line is consensus sequence. Underlines in the consensus sequences represent miR6024, while dash lines under the consensus sequence represent miR6024*. Dots mean nucleotides identical to that of consensus sequence. Dash lines in sequences represent deletions. Sequence name HZ represents tomato Heinz 1706, DM for potato cultivar DM, CA for *C. annuum*, NTA for *N. tobaccum*, NT for *N. tomentosiformis*, NB for *N. benthemiana* and NS for *N. sylvestris*.

To gain insight into the evolution of miR6024 in plants, four members of the miR6024 family were downloaded from miRbase (release 20) including sly-miR6024 from tomato, nta-miR6024 from tobacco, stu-miR6024-3p and stu-miR6024-5p from potato. In addition, whole genome sequences of ten plant species were chosen to identify miR6024 genes using bioinformatic approach (see MM section). The miR6024 sequences were used to BLAST search the ten genomes, and significant hits were found in eight of the ten plant species except in *C. papaya* and *A. lyrata*. However, the flanking sequences of the significant hits in *M. guttatus* and *V. vinifera* could not form hairpin structure, and therefore these two species do not have miR6024. All the six Solanaceae species have the miR6024 sequence and its flanking sequences (miR6024 gene) can form a predicted hairpin structure. The miR6024 gene has no similarity with *I2* homologues except that the miR6024/miR6024* can match the target site in *I2* homologues. Therefore, the miR6024 was not originated from duplication and inversion of *I2* sequences [33]. The 22-nt mature miR6024 was confirmed in a pepper sRNA database (Dr. Li, F., unpublished data). However, point mutations were observed in the miR6024^*^ region of *N. sylvestris* and *N. benthemiana*, and it remains unclear if these mutations affect the biogenesis of miR6024 in the two species (Figure 6). Since miR6024 is present in distantly related species in Solanaceae, we hypothesize that the miR6024 was originated in the common ancestor of the Solanaceae family.

## Discussion

### Divergence of *I2*locus among different Solanaceae species

The *I2* locus is a hotspot for *R-*genes, with several qualitative resistance traits (such as *I2*, *Ty-2* and *Sm* from tomato, *R3, R6* and *R7* from potato, and *L* from pepper) and quantitative resistance traits mapped to this locus. A good understanding of the structure and evolution of this locus will facilitate the cloning and efficient use of *R*-genes from this region.

The *I2* locus contains multiple homologues and spans several megabases on the long arm of chromosome 11 in Solanum. The *I2* homologues in this region form nine well-separated sub-cluster (loci). The sub-loci and their expansion were caused by duplication of individual *I2* homologues, since there was no evidence of large (>10 kb) duplications in this region. The duplication frequency of *I2* homologues might have varied considerably since different species show a large variation in *I2* copy number in different sub-loci. Our data showed that deletions also accounted for part of the *I2* locus divergence between different species (Figure 2).

*I2* homologues from the centromeric part of the *I2* locus were grouped into one clade in the phylogenetic tree of all *I2* homologues from Solanum, while homologues from sub-loci 8 and 9 formed four clades (Figure 4). The five clades are equally related with each other, suggesting that they were differentiated at similar time. After differentiation, the homologues from the centromeric part of the *I2* locus duplicated and generated the sub-loci 1–7.

### The structure of *I2*locus from the *Ty-2*haplotype and *S. habrochaites*LA1777

The resistance gene *Ty-2* against TYLCV in cultivated tomato was introgressed from *S. habrochiates* accession“B6013” [34]. The recombination at the *I2* locus of the *Ty-2* haplotype was shown to be highly suppressed [58]. Recombination suppression is possibly caused by inversion (or other dramatic chromosome change) in this region that may prevent pairing between homologous chromosomes. It remains unclear if such chromosome change also exists in any genotypes susceptible to TYLCV. If such susceptible genotypes are identified, they can be crossed with the *Ty-2* haplotype and the resistance trait can be fine mapped.

Four accessions of *S. habrochiates* were included in this study for analysis of *I2* homologues. The number of *I2* homologues and their sequences seem to vary considerably between different accessions, showing large diversity at this locus. One of the four accessions, accession LA1777, was resistant against strain TYLCV-Cyprus [59] and tolerance to TYLCV strains from Sardinia and Senegal [52, 60, 61]. Different copy numbers of *I2* homologues in the T haplotype (8) and accession LA1777 (13), and no highly similar pairs of genes in the two haplotypes (Figure 3), suggest that they have different *I2* locus, consistent with a previous report that the TYLCV resistance in LA1777 is not encoded by *Ty-2* but by several recessive loci [61].

### Sequence exchanges among *I2*homologues

All *I2* homologues from sub-loci 1–7 in potato were evenly related with each other, similar to the feature of Type I *R*-genes. However, unlike Type I *R*-genes, these homologues showed infrequent sequence exchanges, and they are not extensive chimeras though occasional sequence exchanges were detected among them. The presence of a large number of divergent but evenly related homologues and infrequent sequence exchanges suggested that the *I2* homologues from sub-loci 1–7 of the *I2* locus in potato are Type I *R*-genes. They are mingled with tomato *I2* homologues from sub-loci 1–7 in phylogenetic tree (Figure 4), suggesting that this lineage of Type I genes in potato had existed before the divergence of tomato and potato 7 MYA [53, 54].

The *I2* homologues in sub-loci 1–7 in tomato, on the other hand, were diverged into five well-supported clades (Figure 3). Genes physically linked are not necessarily closely related. For example, the genes located in sub-locus 5 were grouped into two different clades (Figure 3). Members from clade I in Figure 3 showed extensive chimeric structures resulted from frequent sequence exchanges; they are evenly related with each other, with most pair-wise nucleotide identity of 85.6-99.8%. Above features are typical of Type I *R*-genes [15]. In contrast, genes within clades II and IV are highly conserved and each genotype has only one representative. Interestingly, rare sequence exchanges were also found between members from different clades. We conclude that the *I2* homologues from sub-loci 1–7 of tomato has started to differentiate. Some groups have evolutionary patterns of Type I while others have evolutionary patterns of Type II *R*-genes. In contrast, the sub-loci 1–7 from potato did not have such differentiation and maintained as one lineage of Type I genes. Such variation on the organization and evolution of the *I2* homologues between tomato and potato, which diverged from their last common ancestor approximately 7 MYA [53, 54], showed that the evolutionary pattern of *R*-genes may change dramatically within a relatively short evolutionary time.

*I2* homologues from sub-locus 9 of potato were grouped into two different clades (Figure 4). Furthermore, the homologues from these two clades are interwoven at sub-locus 9. Members of different lineages of the *R1* resistance gene are also interwoven in the *R1* resistance gene cluster in potato similar to the organization of the *R1* resistance gene family in potato [62]. The interweaving organization of members from different clades suggests that physical proximity is not the main factor for the differentiation of Type I and Type II *R*-genes [63].

### MiRNAs targeting *I2*homologues

*R*-genes, though critical for the survival of plants, may pose threats to plant fitness if accumulated to a high copy number [29]. To reduce their fitness cost, *R*-genes are often kept at low level of expression. One mechanism for down-regulating the expression of *R*-genes is through miRNAs, which were recently shown to cleave *R*-genes and generate tasiRNAs [33]. In addition to the miR482 family targeting *I2* homologues in potato [33], we identified and confirmed that miR6024 targets *I2* family in tomato. Search of the tomato degradome confirmed the cleavage function of miR6024 and also suggest the cleavage function of tasiRNAs triggered by miRNAs (see Results section). The miR482 family has high expression level in three tissues of potato (leaf, flower and stolon) (five members, with an average of 35,760 reads per million), while miR6024 family has a much lower expression in tomato (leaf, flower and fruit) (2,540 reads per million). MiR6024 was found in distantly related genus (Nicotiana and Solanum) in Solanaceae, and therefore it was most likely originated in the common ancestor of Solanaceae. MiR482 was shown to be an ancient miRNA [64]. However, no cleavage of *I2* homologues by miR482 was confirmed in tomato though the miR482 does exist in tomato genome. Therefore, the collective regulation by miR482, miR6024 as well as tasiRNAs triggered by them may control the expression level of *I2* homologues in Solanaceae, and the down-regulation of the *I2* homologues by these miRNAs may make the expansion of *I2* homologues less costly in fitness. Though silenced by miRNAs, these *R*-genes can exercise their full resistance function when challenged by pathogens such as viruses and fungi, which can suppress the silencing machinary of host plants [65, 66].

## Conclusions

Comparative analysis of the *I2* locus in tomato and potato showed that the *I2* locus contains a large number of homologues with high divergence. Its evolutionary patterns varied considerably between tomato and potato. The *I2* family was targeted by at least two miRNAs, which may play important roles in down-regulation and evolution of this resistance gene family.

### Availability of supporting data

Sequence data described this article can be found in the GenBank data libraries under accession numbers KJ652840 - KJ652901.

## Electronic supplementary material

Additional file 1: Table S1: *I2* homolgoues used in this study. Table S1-1. *I2* homologues indentified in the sequenced tomato genome (version 2.40). Table S1-2. *I2* homologues identified in the sequenced potato genome (version 3_2.1.11). Table S1-3. *I2* homologues from Solanum used in this study. **Table S2.** Sequence exchanges between *I2* homologues. Table S2-1. Sequence exchanges detected between tomato I2 homologues from different clades in Figure 3. Table S2-2. Sequence exchanges detected between potato *I2* homologues from different clades in Figure 4. (XLS 80 KB)

Additional file 2: Figure S1: Dot plot analysis of the *I2* locus in tomato. **Figure S2.** Dot plot analysis of the *I2* locus in potato. **Figure S3.** Distance tree of *I2* homologues from tomato, potato, pepper and tobacco. **Figure S4.** A sequence exchange tract of 162 bp between a Type I and a Type II *I2* homologues. **Figure S5.** The cleavage site of miR6024 obtained through sequencing RACE-PCR products. **Figure S6. A.** Alignment of *T-I2-3* sequence with a cDNA sequence (03330341_71_2) found from a tomato degradome database. **B.** The abundance of *I2*-derived sRNAs. **C.** Cleavage sites of two tasiRNAs triggered by miR6024. The bottom lines of each panel represent 5′ sequences obtained using RACE-PCR. The line in gene *T-I2-3* shows the 3rd tasiRNA, and the line in gene *T-I2-6* shows the 5th tasiRNA. **D.** A degraded RNA retrieved from a degradome database. (DOCX 3 MB)
