# Supplementary material for: The I2 resistance gene homologues in Solanum have complex evolutionary patterns and are targeted by miRNAs
Source: BMC Genomics. 2014 Aug 30;15(1):743. doi: 10.1186/1471-2164-15-743 (PMC4161772; doi:10.1186/1471-2164-15-743)

**Figure S1**. Dot plot analysis of the *I2* locus in tomato. The positions for

nine sub-loci (S-1 to S-9) are shown in both the horizontal and vertical lines. The numbers in brackets after the sub-locus name are the number of *I2* homologues at corresponding positions. No large duplications were found, and duplications were mainly limited to the *I2* sequences but not their flanking regions.
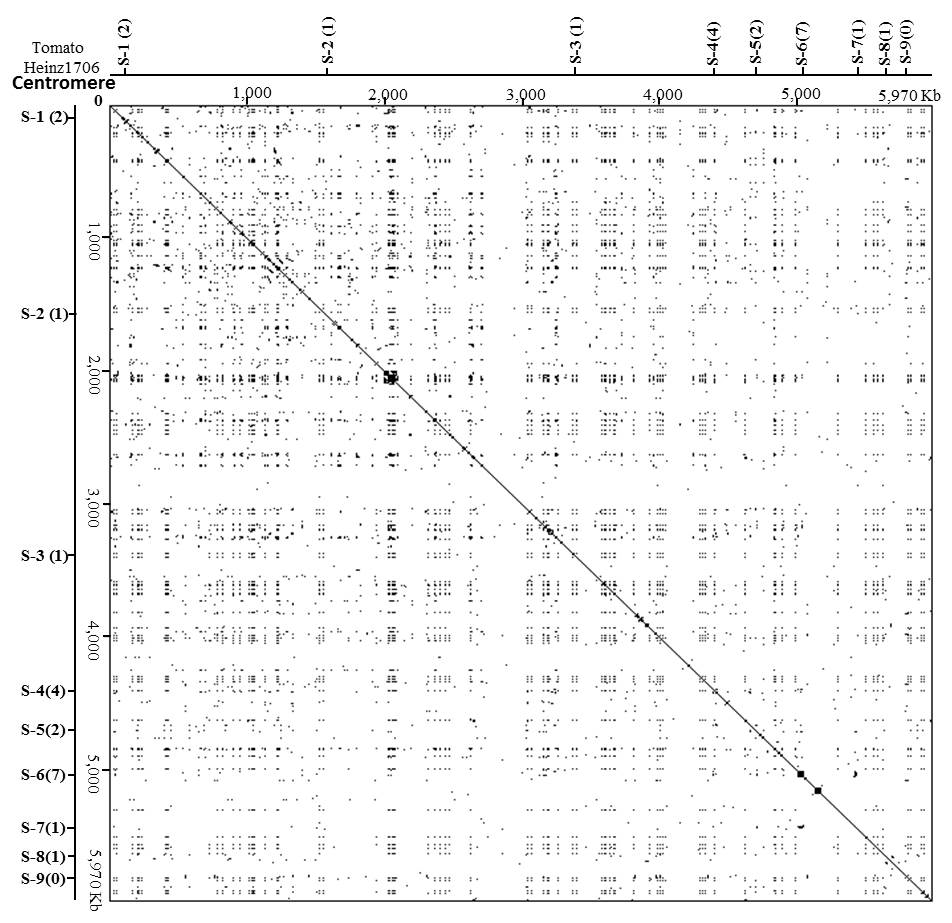


**Figure S2**. Dot plot analysis of the *I2* locus in potato. The positions for nine sub-loci (S-1 to S-9) are shown in both the horizontal and vertical lines. The numbers in brackets after the sub-locus name are the number of *I2* homologues at corresponding positions. No large duplications were found, and duplications were mainly limited to the *I2* sequences but not their flanking regions.
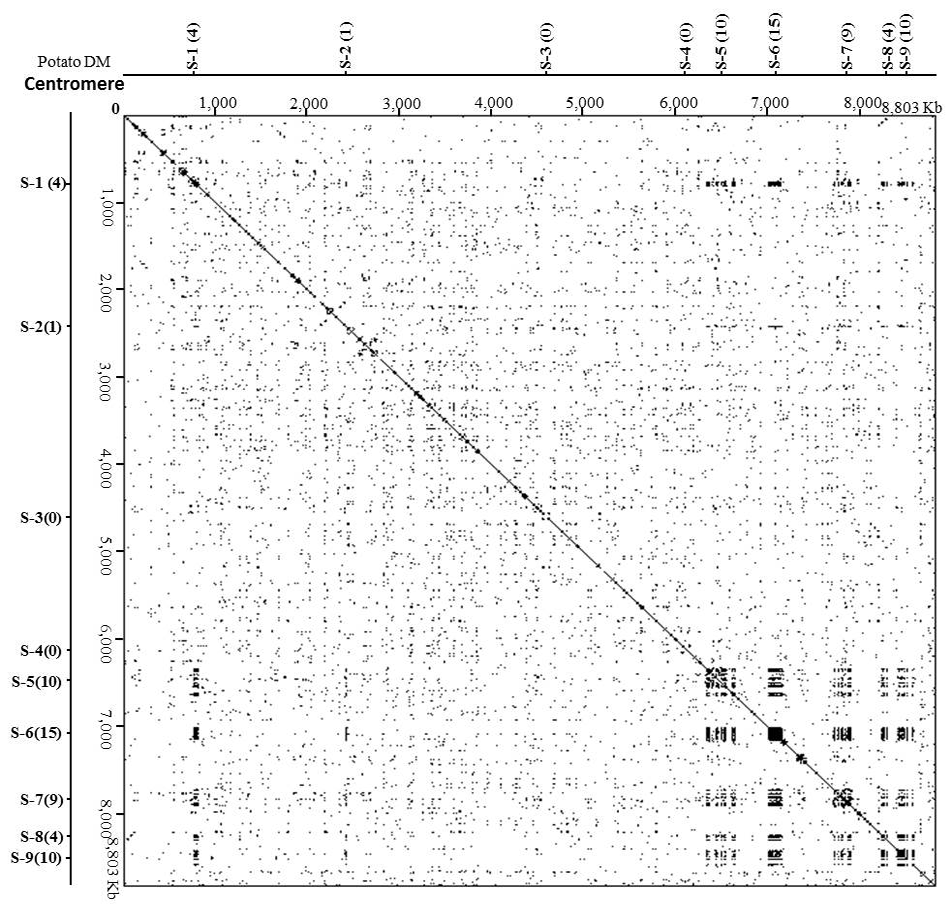


**Figure S3**. Distance tree of *I2* homologues from tomato, potato, pepper and tobacco. The first part of the gene name represents accession/cultivar name, and the numbers in bracket (if any) show gene’s position (sub-locus). Numbers on nodes are bootstrap values, and only values >65 are shown.
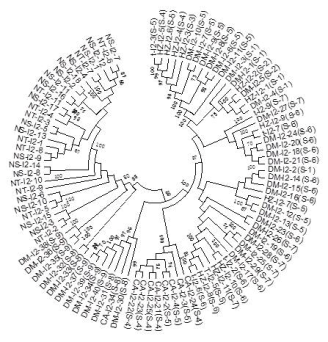


**Figure S4**. A sequence exchange tract of 162 bp between a Type I and a Type II *I2*

homologues. The left panel is sequence names. Sequence exchange tracts between *LA1740-I2-3* (Type II) and *LA1740-I2-5* (Type I) are marked in shadow. Dots represent nucleotides identical to the consensus sequence. The numbers at top are positions in the alignment.


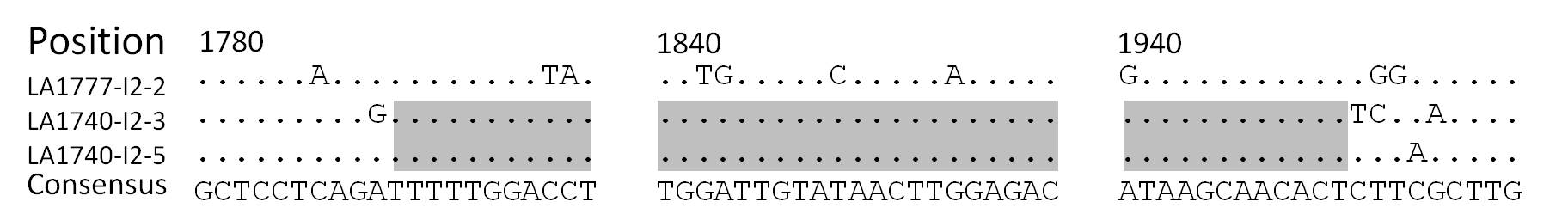


**Figure S5**. The cleavage site of miR6024 obtained through sequencing RACE-PCR products. A. Sequencing chromatography of RACE-PCR products. The left underlined sequences are adaptor and the right underlined sequences are gene primer. B. Alignment of the RACE-PCR products and the sequence of gene *T-I2-3*. All 15 clones sequenced are identical, starting at the predicted position of miR6024 cleavage site as indicated by the arrow.
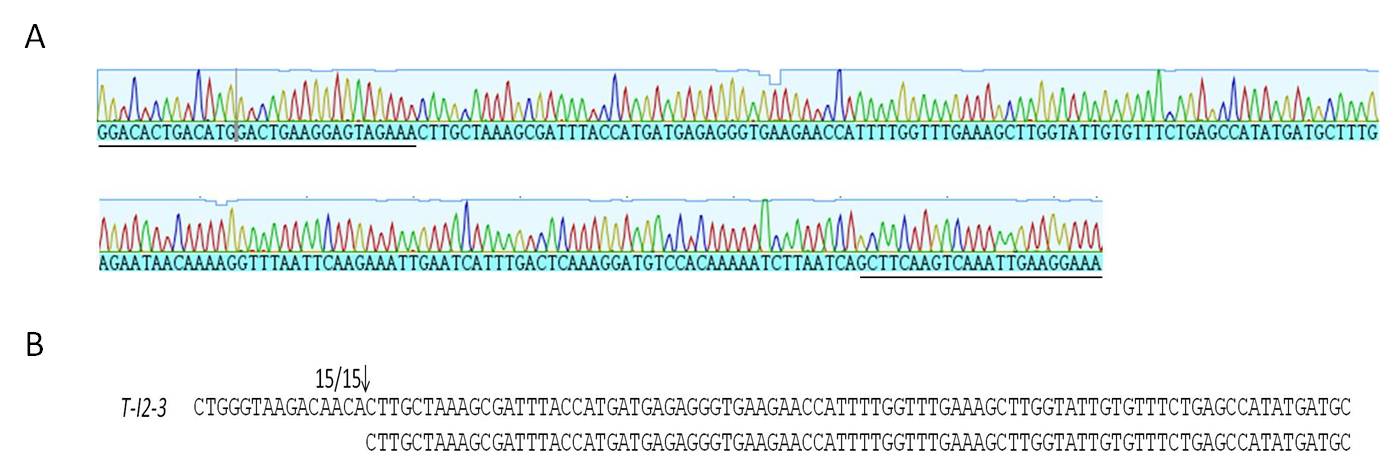


**Figure S6. A**. Alignment of *T-I2-3* sequence with a cDNA sequence (03330341_71_2) found from a tomato degradome database. The top line is miR6024 sequence. The RNA was cleaved at the predicted cleavage site of miR6024, confirming the results of Figure 5. **B**. The abundance of *I2*-derived sRNAs. The x-axis represents a *I2* homologue (*T-I2-3*), and the number are distance from the first nucleotide of sequence (Genbank: KJ652841). The y-axis shows the reads of each sRNA in a library of 5,063,659 tomato sRNAs. Bars above zero are for sRNAs from the positive strand and bars below zero are for sRNAs from the negative strand. The arrow shows the cleavage site of miR6024. **C**. Cleavage sites of two tasiRNAs triggered by miR6024. The bottom lines of each panel represent 5′ sequences obtained using RACE-PCR. The line in gene T-I2-3 shows the 3rd tasiRNA, and the line in gene *T-I2-6* shows the 5^th^ tasiRNA. **D**. A degraded RNA retrieved from a degradome database. The RNA was likely cleaved by the 15^th^ tasiRNA (3′S15).


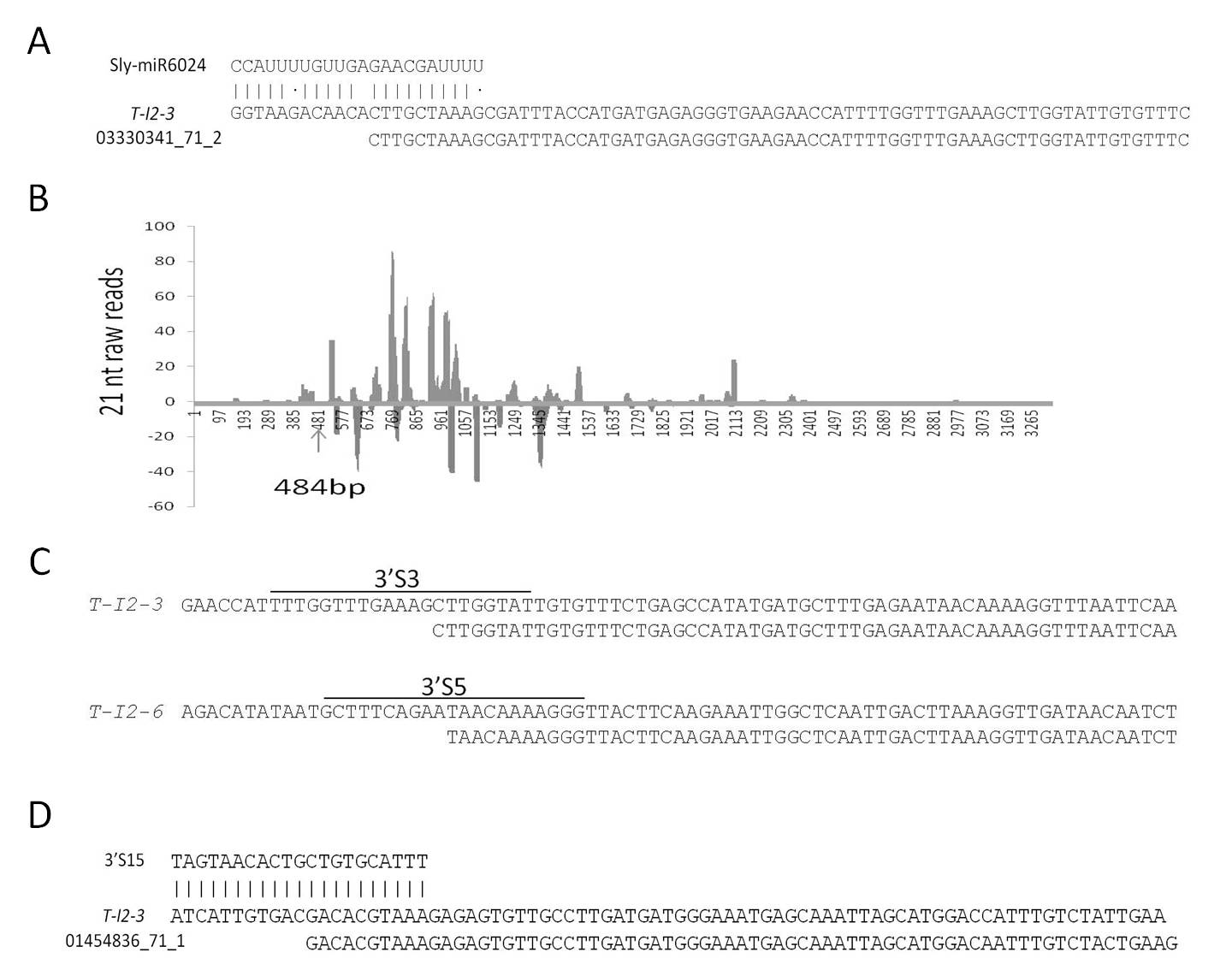

Supplement: Supplementary file 2 — Additional file 2: Figure S1: Dot plot analysis of the I2 locus in tomato. Figure S2. Dot plot analysis of the I2 locus in potato. Figure S3. Distance tree of I2 homologues from tomato, potato, pepper and tobacco. Figure S4. A sequence exchange tract of 162 bp between a Type I and a Type II I2 homologues. Figure S5. The cleavage site of miR6024 obtained through sequencing RACE-PCR products. Figure S6. A. Alignment of T-I2-3 sequence with a cDNA sequence (03330341_71_2) found from a tomato degradome database. B. The abundance of I2-derived sRNAs. C. Cleavage sites of two tasiRNAs triggered by miR6024. The bottom lines of each panel represent 5′ sequences obtained using RACE-PCR. The line in gene T-I2-3 shows the 3rd tasiRNA, and the line in gene T-I2-6 shows the 5th tasiRNA. D. A degraded RNA retrieved from a degradome database. (DOCX 3 MB) [file 12864_2014_6427_MOESM2_ESM.docx]
